# Patient and Clinician Stakeholder Perspectives on a Patient Portal Questionnaire Eliciting Illness and Treatment Understanding and Core Health-Related Values

**DOI:** 10.1089/pmr.2023.0057

**Published:** 2023-11-30

**Authors:** William E. Rosa, Jaime Gilliland, Meghan McDarby, Judith E. Nelson, Anjali V. Desai, Andrew S. Epstein

**Affiliations:** ^1^ Department of Psychiatry, Behavioral Sciences and Memorial Sloan Kettering Cancer Center, New York, New York, USA.; ^2^ Department of Medicine, Memorial Sloan Kettering Cancer Center, New York, New York, USA.; ^3^ Weill Cornell Medical College, New York, New York, USA.; ^4^ Oncology Palliative Care Services, Atlantic Health System, Morristown, New Jersey, USA.

**Keywords:** cancer, communication, patient portal, person-centered care, prognosis, prognostic understanding, technology, values

## Abstract

**Introduction:**

Person-centered communication is foundational to cancer care. In pilot research, a questionnaire eliciting patients' illness and treatment understanding (ITU) and core health-related values (HRV) through the electronic patient portal demonstrated feasibility, acceptability, and efficacy. The aim of this study was to elicit stakeholder feedback to refine the design of the portal-based intervention, remain end-user centered, and optimize future system-wide integration.

**Methods:**

Between April and June 2023, we purposively sampled patients and clinicians from a previous pilot study to participate in a 20–30-minute semistructured interview about their opinions of and experiences with the portal questionnaire on ITU and HRV. An interdisciplinary coding team used a two-phase rapid analysis to identify themes, subthemes, and illustrative participant quotations.

**Results:**

Fourteen patients (mean age = 68 years) and 12 clinicians participated (total *n* = 26). Colorectal cancer was the commonest malignancy (64%) among patients. Clinicians were mostly physicians (50%), nurse practitioners (33%), and registered nurses (17%), with two-thirds having >15 years of experience in their specialty. Analysis generated four themes: (1) clinical utility of questionnaire, (2) barriers to questionnaire implementation, (3) considerations and strategies for modifying the questionnaire, and (4) considerations and strategies for questionnaire implementation. Themes captured key information about incorporating this questionnaire into clinical practice.

**Conclusion:**

Patients with cancer and their clinicians found a portal-based ITU and HRV questionnaire clinically useful to improve multiple aspects of person-centered communication. Participant recommendations about questionnaire timing and sharing of questionnaire responses with the clinical team will inform future questionnaire implementation and scaling in clinical settings.

## Background

Person-centered communication is a two-way process ensuring that patients can adequately understand their illness and treatment options and clinicians know patients as individuals.^1–4^ During this type of communication, clinicians empathically provide expert information while attending to the patient's personhood, illness and treatment understanding (ITU), and core health-related values (HRV). Although person-centered communication is known to positively affect psychological wellbeing^5^ and increase documentation of patients' care goals,^6^ research to date has primarily focused on such communication for patients nearing death,^7–10^ and on specific end-of-life treatment preferences.^11–13^ Furthermore, person-centered communication is fundamental to effective advance care planning (ACP) to promote goal concordance in cancer and other serious illnesses.^14–17^

Oncologic therapies that may improve survival and/or quality of life entail progressively intricate choices and less certain outcomes that require effective communication. Yet several real-world clinical factors (e.g., significant patient volume, acuity, and complexity in health systems with constrained resources) create barriers to person-centered communication,^4^ potentially leading to adverse outcomes and missed opportunities for shared decision making. However, as health systems continue to leverage technology—specifically, patient portals—to improve communication and cancer-related outcomes,^18–20^ there is a pressing need to consider patient and clinician perspectives about the use of portals to improve person-centered communication, both to enhance the clinical utility of portal-focused innovations and to balance factors such as treatment benefits and burdens, survival time, and quality of life.^16^

We have pilot tested a portal-enabled approach called “PERSON” (**P**atient portals to **E**licit essential patient-**R**eported elements of communication **S**upporting person-centered **ON**cologic care)^21,22^ in a sample of gastrointestinal (GI) oncology clinics within our comprehensive cancer center to elicit information about personhood, ITU, and HRV (hereafter, “the questionnaire” refers to the combined ITU and HRV questionnaire items elicited through the portal).^23,24^ This pilot work provided evidence of feasibility and acceptability, while also showing an association between the PERSON approach and higher patient ratings of clinicians' communication quality.^21,22^ In the present study, we explored the perspectives of patients and clinicians who participated in the pilot study for additional feedback about workflow considerations, processes, and portal use related to PERSON. Our aim to identify barriers and facilitators to effective portal-based elicitation of ITU and HRV will assist in refining the design of our questionnaire, ensuring it remains end-user centered, and optimizing future system-wide PERSON implementation.

## Methods

This study was conducted as part of an institutional quality improvement initiative, which the Memorial Sloan Kettering Institutional Review Board reviewed and exempted from informed consent.

### Participants

Between April and June 2023, we invited English-speaking GI patients and clinicians who participated in the PERSON pilot trial to participate in a 20–30-minute semistructured interview on their experience with the questionnaire. In the PERSON trial, all participants received the questionnaire (ITU questions were sent quarterly to 6325 patients, 48 of whom also agreed to participate in an expansion wherein HRV questions were also sent) through the patient portal^21,22^ one week before the second GI clinic follow-up visit, then quarterly. Participants for the current study were purposively sampled among participating clinics with the goal of both patient and clinician demographic diversity.

### Data collection

A qualitative methods specialist (QMS: J.G.) conducted interviews with patients and clinicians in English through Zoom (Zoom Video Communications, Inc., San Jose, CA) or by telephone using a written interview guide (Supplementary Data S1). Participants in each stakeholder group were asked to share their opinions on the importance of collecting information on ITU and HRV, experience with the questionnaire, and perceptions of usefulness and clinical utility. Clinicians were additionally asked about clinical workflow implications for implementation and institutional support. The interview guides (Supplementary Data S1) were originally designed to collect information about ITU and HRV items separately. However, after piloting the guide with three patients, it became clear that patients did not recall specific differences between the two instruments. The patient interview guide was revised to ask about the importance of the two topics individually, while asking about their experience with the ITU and HRV items together. Clinicians also had difficulty distinguishing between ITU and HRV items; thus, we made similar revisions to the clinician interview guide. Interviews were audio recorded for analysis.

### Qualitative analysis

Following the interview, the QMS drafted a detailed summary of participants' responses using the audio recording to include verbatim quotes, based on best practices for rapid qualitative data analysis.^25,26^ Rapid analysis (RA) is a qualitative approach that quickly produces information to inform ongoing implementation efforts, and has been shown to generate results consistent with in-depth text analysis.^26^ RA was conducted in two phases: a “vertical” phase and a “horizontal” phase.^27,28^ In the vertical phase, the QMS reviewed the individual interview summaries, and significant statements were identified based on *a priori* constructs from the interview guide as well as emergent ideas from the interviews. These statements were sorted into categories relating to importance of the information being collected, experience with the questionnaire, perceptions of usefulness and clinical utility, clinical workflow implications, and institutional support. In the horizontal phase, members of the interdisciplinary analytic team (W.E.R., M.M., J.G.) reviewed the statements grouped within each category, across participants, to identify primary themes.

To facilitate this phase, the team created a data matrix, with each row representing an individual participant, and each column representing an analytic category. One matrix was created for each of the two stakeholder groups. The analytic team collaboratively and iteratively reviewed the data to refine the themes and subthemes and to identify illustrative participant quotations. Themes included prominent sentiments within each category, as well as any significant divergences between and within stakeholder groups (e.g., opinions on clinical utility among oncologists vs. patients). Multiple subthemes were identified for each theme. Given participant difficulty in distinguishing ITU and HRV items in the questionnaire, findings were reported in aggregate (i.e., for both ITU and HRV questionnaire items) unless noted.

## Results

### Participant characteristics

Patients (*n* = 14) predominantly identified as White (79%) and male (57%) with a mean age of 68 years. Colorectal cancer was the most common malignancy (64%), followed by pancreatic (14%) and neuroendocrine (14%). The majority of patients were diagnosed with stage III or stage IV disease (36% and 57%, respectively). Clinician participants (*n* = 12) included GI physicians (50%), nurse practitioners (33%), and registered nurses (17%), and two-thirds had >15 years professional experience in their specialty. Tables 1 and 2 provide demographic information for all participants.

**Table 1.** Patient Demographics

Demographics	Patients (***N*** = 14)
Age (mean age in years, range)	68 (55–84)
Gender	
Male	8 (57%)
Female	6 (43%)
Malignancy type	
Colorectal (or small bowel)	9 (64%)
Pancreatic	2 (14%)
Neuroendocrine	2 (14%)
Ampullary	1 (7%)
Stage	
I	0 (0%)
II	1 (7%)
III	5 (36%)
IV	8 (57%)
Race	
White	11 (79%)
Asian	1 (7%)
Black/African American	1 (7%)
Unknown	1 (7%)
Ethnicity	
Non-Hispanic/Non-Latinx	12 (86%)
Hispanic/Latinx	0 (0%)
Unknown	2 (14%)
Marital status	
Married	9 (64%)
Single	3 (21%)
Widowed	1 (7%)
Life partner	1 (7%)
Religion	
Roman Catholic	5 (36%)
None	3 (21%)
Other Christian	3 (21%)
Jewish	2 (14%)
Muslim	1 (7%)

**Table 2.** Clinician Demographics

Demographics	Clinicians (***N*** = 12)
Gender	
Male	2 (17%)
Female	10 (83%)
Race	
White	6 (50%)
Asian	2 (17%)
Black/African American	2 (17%)
Biracial	1 (8%)
Other	1 (8%)
Ethnicity	
Non-Hispanic/Non-Latinx	10 (83%)
Hispanic/Latinx	2 (17%)
Time in practice (mean time in years, range)	15 (4–30)
Discipline	
MD	6 (50%)
NP	4 (33%)
RN	2 (17%)
Demographics	Clinicians (*N* = 12)

### Qualitative themes

Analysis generated four themes: (1) clinical utility of questionnaire, (2) barriers to questionnaire implementation, (3) considerations and strategies for modifying the questionnaire, and (4) considerations and strategies for questionnaire implementation. Themes captured important information about incorporating this questionnaire into clinical practice. Responses from all participants supported and clarified each theme and each theme's properties (Table 3).

**Table 3.** Major Themes, Qualitative Findings, and Illustrative Quotes

Themes	Qualitative findings and illustrative quotes
Clinical Utility of Questionnaire	Increases opportunities for patient involvement and goal-concordant care “When someone's asking you questions like that, it gives you a message that they care and care personally about you. And I think that's the most important part, that a patient feels like they're not just there and part of a huge process, but they're really important to whoever is working with them.”—Patient “It's very important. If something happens, we can show the family [the responses] in case the patient cannot answer by themselves what the patient would like to have done, what were their thoughts about their care. So, it makes it an easier conversation with the family.” —Clinician “Anchors” conversations and communication between patients and providers “[The questions] ‘what does living well mean to you’ and ‘what gives you strength’—those are really great ways for us to anchor some of our discussions about therapies people are on, the chances of it working, and side effects, and how it might impact their ability to do some of those things [that are important to them].” —Clinician “We always talk when I go in for my follow ups, we talk a lot. And I'll express some things that were on the questionnaire.” —Patient Recognizes necessity of questions and related discussions even if content is upsetting or difficult “It affected me, not only for the moment I was talking about it, but I was thinking about it for the next few days and I was a little down after that. Is it good or not good? I think it's good to talk about it. […] I think I have to face life.”—Patient “The population that might struggle the most with these are the ones that get angry or frustrated about having these conversations and then this is just another reminder when they're not in the doctor's office, about the bigger picture of what's going on. Sometimes that can be tough for our patients who are really struggling. I feel like, when they're away from the clinic or the chemo space, mentally they may be in a different place, and then they get this prompt from the portal and [they go to fill it out] and it's like, ‘ugh, it's asking me again what I think about big picture related to my disease.’ The flip side of that is that it's good to give these patients some space to think about this outside of the doctor's office.”—Clinician
Barriers to Questionnaire Implementation	Continuity between clinics/inpatient and outpatient limits' integration “I think having a separate tab is great, the in-patient team can go to that, the out-patient team can go to that, ICU team can go to that.”—Clinician “[It's an] irritating situation when you have a sick, complex patient and the patient comes into the hospital and a physician who doesn't know patient asks me to come have goals of care discussion when all of that information is in notes.”—Clinician Clinician-specific limitations in terms of time “[Portal-based elicitation] is one of the steps towards addressing the time issue in the clinic visit. This is a good way to start taking some of the time away from the visit[…] you're already resourced with the data and you don't have to necessarily extract all that data during your clinic visit.”—Clinician “Still not enough time in the day to do this well. 15-minute clinic visit, nurses have injection clinic in addition to toxicity checks and other things. What falls by the wayside is this type of important information because it is not urgent.”—Clinician Lack of institutional support “I feel like there are certain groups within the institution that are supportive of [collecting and utilizing this type of data]. As an institution, we have a lot of other things that we need to be doing and I wish we had more time to do this.”—Clinician “If [a patient has] low health literacy and we've talked about this 3 times already, I get a 15-minute time slot no matter what. So, what's the resource? There is awareness but that doesn't fix the situation.”—Clinician
Considerations and Strategies for Modifying Questionnaire	Provide concrete explanation on the measures to patients about what the measures are for and how they will be used (“prime patients” about questionnaire and let them know they're coming) “I didn't quite understand how it was being used. I guess I assumed[…] that it was being aggregated as well as being read individually.”—Patient “These kinds of questions are new for patients, and some felt like, ‘what does this mean? Is there an underlying meaning here? Is this because my disease has poor prognosis?’ We need to relay that we ask all patients these questions, regardless of disease type or stage, explain we want to understand them as an individual and what's important to them. Communicate that we want to document it for the care team.”—Clinician Provide structured guidance to patients about how to complete questionnaire “At the beginning, there could have been some type of lead-in […] that expresses what it's looking for. ‘We're looking for your most in-depth feelings’, ‘we're looking for you to tell us how you really feel deep inside’—those cues to focus people in that direction.”—Patient
Considerations and Strategies for Questionnaire Implementation	Modality of how people respond is important to consider, but overall, using the portal is promising and agreeable to patients “I thought that [responding via the portal] was perfect. You got to reflect and give honest answers as opposed to being in a conversational setting.”—Patient “In clinic, you're kind of put on the spot and you're rapid-firing answers. Sometimes the answers might not be as reliable as doing it at home on my own time.”—Patient Timing of survey administration and follow-up conversations “I think it's a little redundant to think your outlook would change every 4 months. Or maybe it would, but I haven't experienced that yet.”—Patient “[These discussions should] not [happen] the first visit—the first visit when he tells you, ‘I don't think I can cure you’ when other people have been saying, ‘ah this ain't so bad’… that's a real kick in the teeth. You and whoever you're with, after going ‘uh oh’, takes some time to digest that. So, not the very first meeting but maybe the second or possibly the third meeting.”—Patient Make survey more inclusive and more individualized “Sometimes [the questions] are difficult emotionally. The reality of my life before having cancer and now has changed so completely in terms of my outlook, that that's what it reminds me of.”—Patient “The one that made me really emotional was about talking to anyone in my family about what I want to happen at the end. I know it's important to talk about but thinking of it this way makes it almost final.”—Patient Streamline for clinicians/workflow considerations “If the nurses could carve out just a little time to say, ‘I went through these values [questions] and just a couple of things to note.’ Whether it's in your MDR, in clinic that day[…]”—Clinician “I think that I probably should have been a little bit more focused on trying to make sure that, for my own purposes, [the responses] actually fed back to the clinic as opposed to just having the nurses and NPs talk to the patients about the surveys. […] That would have been helpful, for me to maybe try to document that, to understand that it's yet another check to make sure that the patient and I are aligned on what we think is going on.”—Clinician

#### Theme 1: Clinical utility of questionnaire

Patients and clinicians overwhelmingly agreed the questionnaire was useful and clinically meaningful, especially in highlighting the “whole personhood” of patients, creating opportunities for patients to receive tailored, person-centered care, and increasing opportunities for concordance with patient goals. For example, patients reported that when their clinician understands their HRV (per questionnaire responses), their treatment recommendations are subsequently customized and individualized. Participants also shared that the questionnaire invited patient participation in care by considering their emotions, and sometimes supported caregiver involvement in conversations (e.g., can aid caregivers in understanding patient care preferences or provide opportunities for caregivers to provide input on patient care).

Participants described the questionnaire as a way to “anchor” conversations and overall communication between patients and clinicians. For example, patients and clinicians stated that questionnaire responses supported follow-up conversations or sparked ideas for topics to cover during appointments. Importantly, all participants noted the questionnaire helped serious illness conversations occur more frequently than they would naturally arise, and that questionnaire information gave clinicians a greater sense of patient preferences for information delivery during those conversations (e.g., the volume and specificity of information that should be delivered). Clinicians noted that questionnaire responses offered insight about patients' understanding of clinicians' previously communicated information.

Some respondents agreed that completing the questionnaire could be anxiety-provoking, but that the information is critical to discuss and best support the patient. Most patients stated that they did not experience negative emotions when completing the questionnaire, but rather, it helped them feel included in important care discussions.

#### Theme 2: Barriers to questionnaire implementation

Patient participants did not speak specifically to implementation barriers. However, clinician respondents pointed to three primary barriers to implementing the questionnaire in clinical practice. Clinicians noted a lack of continuity of care and information flow between clinics, inpatient care settings, and other outpatient care settings, which they believed limits the successful integration of the questionnaire into care delivery. Clinicians especially noted that without effective information flow between inpatient and outpatient settings, patients' preferences and responses may not be accurately communicated—or communicated at all—across settings. Clinicians noted a need for improved awareness of the questionnaire, as well as associated conversations among other clinicians across the institution.

Clinicians also described the challenge of integrating the questionnaire data into their clinic workflow due to time constraints; sometimes they were simply “too busy” to incorporate the questionnaire and associated conversations. For example, clinicians described sometimes having as little as 15 minutes with each patient, then stated that discussion of patient information from the questionnaire could be “cut” in favor of more time-sensitive patient needs, such as immediate symptom management. Clinicians also discussed the challenge of needing to interrupt or stop HRV-related patient conversations due to limited appointment time, at the expense of reducing participation for patients who are particularly engaged in the discussion of their HRV.

Clinicians described lack of institutional support as an implementation barrier, noting that full integration of the questionnaire would require a shift across disciplines—including physicians, nurses, and social workers—to actively prioritize the questionnaire and the conversations it supports. Clinicians further noted that without overarching institutional support, it could be difficult for even the strongest champions to actively integrate the questionnaire or prioritize the “face time” required for effective HRV conversations. Participants pointed to the importance of participation and endorsement of the questionnaire “from the top.”

#### Theme 3: Considerations and strategies for modifying the questionnaire

Participants addressed several directions for improving the questionnaire. Participants, primarily patients, described the need for concrete explanations about the purpose of the questionnaire to the patient (e.g., what the questionnaire is for, how questionnaire data will be used). Several patients needed help to complete the questionnaire because they lacked clarity about the intent of the questions. Patients also advised that the questionnaire could include additional language about the nature of the questions (e.g., they are optional, clinicians ask these questions of all patients).

Participants recommended that questionnaire language should be modified to provide structured guidance about how to complete it. For example, some patient participants reported confusion about how to answer open-ended questions on the questionnaire and what type of information was being requested. Participants noted that, especially in a major cancer center where patients are asked to complete many questionnaires and surveys, clarification and details about overall intent would help them differentiate from other questionnaires.

#### Theme 4: Considerations and strategies for questionnaire implementation

Participants noted that very few patients broached the questionnaire with their clinicians in-person, thus clinician awareness of the questionnaire remained mixed. As a result, participants provided guidance to develop specific strategies for staged implementation. Participants noted that using the patient portal was generally an effective modality for patients to complete the questionnaire. Patients, specifically, stated that the portal gave them the opportunity to review the questions, respond in advance of their clinic visit, and process-related information at home before their appointment. Clinicians shared that receiving patient responses through the portal reduced time that would be needed for them to answer questions during the appointment. Although a few patients reported that they were inclined to give shorter responses when completing the questionnaire on their smartphone (vs. on a computer), there was significant agreement among patients and clinicians that completing the questions through the portal supported patients in having time to reflect and provide honest answers, without the pressure of responding during an actual appointment.

Respondents emphasized the importance of the timing of questionnaire administration and possible follow-up conversations. Patients and clinicians pointed to the benefit of multiple, repeated questionnaires, spaced in a way that would allow changes in prognosis and treatment status to inform conversations during appointments. Many participants believed that spacing the questionnaires apart by 6–12 months would be most useful, as every 3 months felt too repetitive. Patients recommended offering the questionnaire after major changes to care (e.g., treatment change, post-scan disease progression) would be appropriate.

Participants also offered suggestions for making the questionnaire more inclusive and even more individualized, including by cancer type, treatment stage, and patient age. For example, a small number of patients endorsed distress upon completing the questions, and clinicians noted that some patients might indeed experience such distress. Participants therefore suggested addressing potential distress as part of the survey. Respondents also pointed to the need to offer alternative modalities of questionnaire delivery for patients not using the portal.

Clinicians offered recommendations to streamline the process for workflow purposes. For example, clinician participants noted that practicing clinicians who understood the questionnaire and were aware of its integration into the patient portal would be more motivated to incorporate them into their typical workflow. To achieve increased clinician awareness and greater workflow integration, participants suggested providing clinicians with email or chart reminders around the patient's second or third appointment with the clinician. Participants also recommended a nurse triage approach, wherein nurses would review patients' responses in the portal in advance of the follow-up clinic visit and communicate results to the oncologist. Clinician participants also provided suggestions about how to distribute the questionnaire information during an appointment (e.g., providing clinicians with a printed copy or email with embedded responses to reference during the appointment).

## Discussion

This qualitative study obtained patient and clinician perspectives on the workflow, processes, and usability of the PERSON questionnaire that elicited both patient ITU and HRV. Findings suggest that questions about ITU and HRV presented through the patient portal can be clinically useful in achieving goal-concordant care while improving overall clinical communication and helping patients to feel cared for, regardless of available treatment options. Insight from patients and clinicians provided our team with a data-based roadmap to further refine our portal questionnaire, in terms of timing and user friendliness, the infrastructure within which the questionnaire is delivered, and opportunities to strengthen both system-level and interprofessional understanding of intervention aims and integration.

Although guidelines suggest that oncology clinicians consistently incorporate person-centered communication throughout clinical encounters,^4^ patients in our study noted that ITU and HRV questions and conversations were usually not occurring in clinical settings independent of the pilot strategy, and furthermore, the introduction of the PERSON questionnaire was the first time clinicians explored in-depth key elements of their personhood. Some patients expressed surprise at being asked this type of information, either expecting a sole clinical focus on biomedical interventions or not understanding how these aspects of their individuality were relevant to their treatment plan. However, many participants shared that the PERSON questionnaire helped them to “feel heard and understood,” which is considered a patient-reported measure of care quality.^29,30^ Thus, our current stakeholder data suggest that the portal-elicited ITU and HRV questions will have positive and measurable impact on care quality outcomes as the PERSON approach is refined and scaled institutionally.

The PERSON questionnaire is positioned to become a reliable intervention to promote goal-concordant and person-centered care delivery through portal-based ACP. HRV elicitation is a core component of ACP^31^ and shared decision making,^32,33^ and is distinct from goals of care discussions, which have been less clearly defined^34^ and tend to focus on treatment planning or care preferences related to an episode of care or care transitions.^35–37^ Addressing HRV can shed light on more self-defined and stable aspects of personhood beyond end-of-life wishes, including a patient's sources of strength, hopes, concerns, and their definition of acceptable quality of life.^38–40^ Despite mixed empirical evidence on the associations between ACP and cancer care at the end of life, the communication aspects of ACP tend to have more influence than other components, such as documents, on related outcomes.^41^ Using the PERSON approach to support person-centered communication may support clinicians in leveraging technology and implementation science methods to facilitate iterative ACP throughout the lifespan and disease course.^14,15^

For instance, participants in our study recommended that regularly administering the PERSON questionnaire ∼6–12 months apart through patient portal would support patient reflection and ongoing discussions with clinicians in the context of prognostic and treatment changes. Similar approaches to early nurse-led palliative care integration have reflected core aspects of our intervention, including leveraging interdisciplinary skills and practice priorities with goals of facilitating cross-team collaboration and communication, integrating patients' personal narratives, and moving from a disease-focused model to the promotion of living well with cancer.^42^

Results should be considered in the context of the following limitations. Ours was a relatively limited sample that hinders generalizability. Despite the smaller sample size, we reached data saturation within the allotted number of interviews,^43^ allowing the coding team to identify salient themes. Although we purposively sampled patients and primary oncology clinicians, future research should include the perspectives of caregivers who may assist patients with portal completion of ITU and HRV questionnaire and are active participants in clinic encounters where the bulk of communication occurs. Most patient participants were also White and Christian, limiting the inclusion of more diverse group perspectives. Additional research should focus on capturing the experiences of minoritized groups related to ITU and HRV. Other interdisciplinary clinicians who find ITU and HRV data beneficial to fulfilling their professional responsibilities (e.g., social workers, chaplains, palliative specialists) should also provide future insights to best translate portal elicited information into care delivery.^23^ In addition, more than 90% of the patients interviewed were diagnosed with stages III or IV disease.

Since person-centered communication should ideally begin at the time of cancer diagnosis, it will be important to gain further insights from patients earlier in their disease process, facing different and possibly less complexity in decision making and treatment options. Finally, future elicitation of broader patient portal implementation barriers from the patient perspective would assist to identify other elements not captured in the current study (e.g., no portal or technology access, log in issues, technological literacy) and to further refine our intervention.

In sum, the patients and clinicians who participated in our intervention shared positive feedback that supported its clinical utility and actionable guidance to further strengthen our approaches to integrated portal questionnaire of ITU and HRV. Our ongoing research is addressing barriers to implementation that participants noted, including the timing of portal questionnaire delivery and workflow streamlining for clinicians. This innovative approach to portal-elicited ITU and HRV data presents a promising intervention to enhance clinician–patient relationships throughout the cancer trajectory, use technology to improve person-centered communication, and promote tailored, value- and goal-concordant cancer care in the context of emerging therapeutic and scientific advances.

## Supplementary Material

Supplemental data

## Abbreviations Used

ACP: advance care planning
GI: gastrointestinal
HRV: health-related values
ITU: illness and treatment understanding
RA: rapid analysis
