# Supplementary material for: Patient and Clinician Stakeholder Perspectives on a Patient Portal Questionnaire Eliciting Illness and Treatment Understanding and Core Health-Related Values
Source: Palliat Med Rep. 2023 Nov 30;4(1):316–25. doi: 10.1089/pmr.2023.0057 (PMC10712361; doi:10.1089/pmr.2023.0057)
Supplement: Supplemental data [file Suppl_DataS1.docx]

**Supplement. Patient and Clinician Interview Guides.**

**Portal-Based Questionnaires to Learn About Patient Understanding of Care and Communication and Their Health-Related Values - Patient Interview Guide**

[For patients doing care and communication only]

Thank you for agreeing to participate in this study. Since mid-2022, MSK has been sending a brief questionnaire to patients with gastrointestinal cancers via the portal that asks you about your understanding of your illness and the intent of the cancer treatment, based on the most recent conversations with the oncologist. We are conducting these interviews to learn about your experiences with and views about this process. This interview will take about 20-30 minutes.

We know MSK sends out a lot of different questionnaires, but our conversation will focus on the one that asks about your understanding of your illness and treatment.

Would it be helpful for me to review the questions with you to jog your memory?

[For patients doing care and communication and values questionnaire]

Thank you for agreeing to participate in this study. Since mid-2022, MSK has been sending a brief questionnaire to patients with gastrointestinal cancers via the portal that asks you about your understanding of your illness and the intent of the cancer treatment, based on the most recent conversations with the oncologist. We have also begun investigating using the portal to get a better sense of patients’ values. By values, we mean peoples’ core health-related values – in other words, what’s most important to them during their care. We are conducting these interviews to learn about your experiences with and views about these two processes – the care and communication questionnaire and the values questionnaire. This interview will take about 30-45. We know MSK sends out a lot of different questionnaires, but our conversation will focus on the ones that ask about your understanding of your illness and treatment, and your values.

Would it be helpful for me to review the questions with you to jog your memory?

[For all patients]

We are interested in learning your thoughts and opinions, so anything you share will be very informative. There are no right or wrong answers to any of the questions I’m going to ask you today. You can skip any questions you don’t want to answer, and you can end the interview at any time, for any reason. In order to make sure we get through all the questions today, I may move the conversation forward.

Do you have any questions so far?

I am asking participants for their consent to audio record this interview. This is to allow me to concentrate on you without having to take notes, and to allow our research team to carefully summarize participants’ responses later. Your responses to this interview will be deidentified, which means that your name will not appear anywhere on paper or on our audio recording. Do I have your permission to start audio recording our conversation now?

__ _________ (Interviewer initials here if participant agrees).

**First, I’d like to ask your opinion about the practice of discussing illness understanding and patient values as part of care delivery.**

1. Do you think it is important to discuss patients’ values as part of care delivery? Why or why not?
   1. How does discussing values make you feel?
      1. Probe: More like a person and less like a number?
   2. Have you ever had a conversation about your values with a healthcare provider that is not tied to a questionnaire? Tell me more about that.
      1. What worked well?
      2. What would you have changed?
   3. Do you think these kinds of conversations are valuable? Why or why not?
   4. When do you think these conversations should happen? (e.g., soon after beginning care, later in the process of care, etc.)
2. Do you think it is important to ask patients about illness and treatment understanding? Why or why not?
   1. How does discussing illness and treatment understanding make you feel?
   2. How do you think this might impact care delivery?

**Next, I’d like to ask you a few questions about your experience receiving and responding to the questionnaires.**

1. What was your experience like receiving the questionnaires?
   1. Were they explained to you before you filled them out? Tell me more about that.
2. What was your experience like responding to the questionnaires?
   1. Did you involve anyone else – e.g., family, caregiver, or friend – in responding?
      1. [If yes]: Tell me more about their involvement.
   2. What did you think about the format of the questionnaires?
      1. Multiple choice, free text fields, etc.
3. What do you think of the questions that were asked?
   1. Were any questions unclear, confusing, or difficult to answer? Tell me more about that.
   2. Should any changes be made to the questions asked? Tell me more about that.
4. What feelings were brought up by completing the questionnaires?
   1. Were any questions upsetting? Tell me more about that.
   2. Did any questions cause worry about your prognosis? Tell me more about that.
   3. Did you find the questionnaire burdensome in any way? Tell me more about that.
   4. Did you feel pressure to answer questions a certain way or that a certain answer was required or expected?
5. What did you think about responding to the questionnaires through the portal?
   1. Would you have preferred to respond through another method such as over the phone or in person? Why or why not?

**Now I’d like to ask you a few questions about your thoughts regarding the usefulness of the questionnaires.**

1. Do you think the questionnaires ask for useful information? Why or why not?
   1. *Probe about second questionnaire based on patient’s response*
2. Did you ever discuss the questionnaires with anyone on your care team? (e.g., nurses, oncologist)
   1. [If yes]: Who brought the questionnaires up? You or the clinician?
   2. [If yes]: Tell me more about that conversation.
      1. [If clinician brought up]: Did you like that they brought it up?
      2. [If patient brought up]: Would you have preferred the clinician to bring it up?
      3. What, if anything, did you like about the conversation?
      4. What, if anything, would you have changed about the conversation? (e.g., how the subject was brought up, how the information was handled, when/where the conversation happened, etc.)
   3. [If no]: Would you have liked to discuss the questionnaire with someone on your care team? Why or why not?
      1. [If yes]: Would you have preferred the clinician to start the conversation?
3. How would you like the information in your responses to be used?
   1. What are your thoughts/feelings about posting your responses in your electronic medical record so that the various people involved in your care can all see them (e.g., oncologists, nurses)?
4. What are your thoughts/feelings about receiving the questionnaires every few months, on an ongoing basis?
   1. Is it a good idea to check in with you every few months (i.e., what is happening with the cancer, what is the purpose of the treatment, your values), or is once enough?
5. Is there anything else you’d like to share about your experience with these two questionnaires that I didn’t ask you about?

**That’s all the questions I have for you today. Thank you so much for your participation.** [STOP AUDIO RECORDING]

**Portal-Based Questionnaires to Learn About Patient Understanding of Care and Communication and Their Health-Related Values - Clinician Interview Guide**

[for clinicians only responding about care and communication]
Since mid-2022, MSK has been sending a brief questionnaire to patients with gastrointestinal cancers via the portal that asks patients about their understanding of their illness and the intent of the cancer treatment, based on the most recent conversations with the oncologist. This questionnaire – called the “Understanding of Care and Communication” questionnaire or “ITU questionnaire” - is initially sent soon after the patient first visits with the oncologist, and then again, every four months. Patients respond to two questions in their own words: 1) What are you expecting to happen with the cancer, and, if they indicate they are receiving treatment for their cancer, 2) What do you think is the purpose of your treatment? The other questions ask patients to select from multiple choice answers how likely they think their treatment will 3) cure the cancer, 4) prolong their life, and 5) help them with problems related to the cancer. Over a two-year period, we have found that almost all patients receiving treatment provide responses to this questionnaire on the portal. Their responses are displayed on the “Patient Values Tab” in CIS. We are conducting these interviews to learn about your experiences with and views about this process. This interview will take about 20-30 minutes.

[for clinicians responding about care and communication and values elicitation]

Since mid-2022, MSK has been sending a brief questionnaire to patients with gastrointestinal cancers via the portal that asks patients about their understanding of their illness and the intent of the cancer treatment, based on the most recent conversations with the oncologist. This questionnaire – called the “Understanding of Care and Communication” questionnaire or “ITU Questionnaire” - is initially sent soon after the patient first visits with the oncologist, and then again, every four months. Patients respond to two questions in their own words: 1) What are you expecting to happen with the cancer, and, if they indicate they are receiving treatment for their cancer, 2) What do you think is the purpose of your treatment? The other questions ask patients to select from multiple choice answers how likely they think their treatment will 3) cure the cancer, 4) prolong their life, and 5) help them with problems related to the cancer. Over a two-year period, we have found that almost all patients receiving treatment provide responses to this questionnaire on the portal. Their responses are displayed on the “Patient Values Tab” in CIS. We have also begun investigating using the patient portal for patients to report not just their understanding of illness/treatment but also their health-related values what is most important, concerning and prioritized by patients during their care.

We are conducting these interviews to learn about your experiences with and views about both these processes. This interview will take about 30-45 minutes. Part 1 will focus on the care and communication questionnaire, and part 2 will focus on values questionnaire.

[For all clinicians]

We are interested in learning your thoughts and opinions, so anything you share will be very informative. There are no right or wrong answers to any of the questions I’m going to ask you today. You can skip any questions you don’t want to answer, and you can end the interview at any time, for any reason. In order to make sure we get through all the questions today, I may move the conversation forward.

Do you have any questions so far?

I am asking participants for their consent to audio record this interview. This is to allow me to concentrate on you without having to take notes, and to allow our research team to carefully summarize participants’ responses later. Your responses to this interview will be deidentified, which means that your name will not appear anywhere on paper or on our audio recording. Do I have your permission to start audio recording our conversation now?

__ _________ (Interviewer initials here if participant agrees).

**PART 1: Care and Communication Questionnaire**

1. Tell us about your experience with the ITU questionnaire (Understanding Care and Communication) that is sent to patients quarterly via the patient portal.
   1. How, if at all, did you and your team incorporate the ITU questionnaires into your workflow? (Probes: Clinic prep meetings, discussions with patients in clinic, team discussions after clinics)
      1. [If affirmative response that questionnaire was incorporated]: Did you view the responses personally or hear about them from another member of the team? Tell me more about that.
         1. How do you usually access patient information on the portal?
      2. [If affirmative response that questionnaire was incorporated]: Tell me more about those interactions/conversations.
         1. What went well?
         2. What didn’t work?
         3. Who usually brought up the conversation? You or patients/families?
   2. How do you prefer to be notified that patients have responded and have ready access to the responses at the time of the visit?
      1. During the pilot, notification was sent via email. What did you like / not like about this?
2. Did you find value in the ITU questionnaires of patients via the patient portal?
   1. Why or why not?
   2. What, if anything, could be done to improve the value?
3. What challenges, if any, did you have with the ITU questionnaires of patients via the patient portal? Tell me more about that.
   1. Probes: didn’t have ready access, burdensome, time consuming, seemed distressing to patient, difficult to talk about at visit
   2. Overall, do you feel the value of the ITU questionnaires outweighs the challenges?
4. Are there particular patient populations for whom ITU questionnaire would be most valuable? (if any, why them)
   1. Probe: advanced cancer, advancing cancer, patients wanting detailed medical info, non-English speakers, minority populations
5. What improvements, if any, do you think are needed to the ITU questionnaires of patients via the patient portal?
6. [If participant is only doing part 1]: Do you feel institutionally supported in collecting and utilizing this information from patients?
   1. Why or why not?

*If clinician is not participating in part 2:* **That’s all the questions I have for you today. Thank you so much for your participation.** [STOP AUDIO RECORDING]

*If clinician is participating in part 2:* **That’s all the questions I have for you about the care and communication questionnaire. Now we’ll transition to part 2 of the interview and talk more about portal-enabled values elicitation.**

**PART 2: VALUES QUESTIONNAIRE VIA PORTAL**

How important do you think eliciting patient values is to overall patient care planning?

1. Prior to sending these questions via portal, how did you incorporate this into care discussions, if at all?
2. Tell me about your experience with the values questionnaire of patients via the patient portal.
   1. How did your team incorporate it into your workflow? (e.g., before/in clinic, documentation after seeing patient)
   2. How did you utilize the responses to the values questionnaires with patients?
      1. [If affirmative response that questionnaire was incorporated]: Tell me more about those interactions/conversations.
         1. What went well?
         2. What didn’t work?
         3. Who usually brought up the conversation? You or patients/families?
      2. Is there anything that might help facilitate these conversations with patients? (e.g., discussion guides, tip sheets)
   3. Did you view the responses personally or hear about them from another member of the team? Tell me more about that.
   4. How do you want to be notified that patients have responded and have ready access to the responses at the time of the visit?
      1. During the pilot, notification was sent via email. What did you like / not like about this?
3. Did you find value in the values questionnaires of patients via the patient portal?
   1. Why or why not?
      1. Are there any specific examples you can share?
   2. What, if anything, could be done to improve the value?
      1. Probes: Include links in the notification materials to the oncology team with any guidance such as on the 1) MSK Patient Values Tab (Desai et al – JCO Oncol Pract, Desai et al JMIR), 2) resources like the A-N-P paper published by MSK Supportive Care (Kramer D et al, Palliative Supp Care, 2022) on how nursing professionals can respond to various emotions that may arise when discussing patient values, 3) communication skills resources the MSK Supportive Care Service developed during the COVID surge, or 4) the “Oncolo-GIST” approach (Epstein A et al, J Pain Symptom Management, 2020) for oncologists to discuss prognosis in patients where such is clinically indicated and/or desired by the patient? 5) Other?
4. What challenges, if any, did you have with the values questionnaires of patients via the patient portal? Tell me more about that.
   1. Probes: access to responses, burdensome, time consuming, distressing to patients, difficult to communicate about during visit
   2. Overall, do you feel the value of the values questionnaires outweighs the challenges?
5. In the past, values discussions for some patients were conducted 1-on-1 between patients and their oncology team’s nurse. Comparing this with patients reporting values via portal questionnaire, what do you see as the advantages of one approach over another?
   1. What do you see as the disadvantages?
6. Do you recall being provided patients’ prior responses to questions about how they like to receive information (e.g., in detail or not, and with family present or not), and what they understood about their illness and the intent of its treatment?
   1. [If yes]: Do you think providing this kind of information together for oncology teams was helpful?
      1. Why or why not?
      2. Is there any other patient-specific data that might make communication easier?
      3. When do you think this information should be shared?
         1. Probes: Before or after scans, before or after visits
      4. How should this data be presented to oncology teams?
7. How would such data best be integrated into the EHR?
   1. Probes: Values Tab, Progress Notes, Goals of care notes
8. Do you feel institutionally supported in collecting and utilizing information related to illness and treatment understanding and values from patients?
   1. Why or why not?
9. Is there anything else I didn’t ask you about related to either questionnaire that you want to share?

**That’s all the questions I have for you today. Thank you so much for your participation.** [STOP AUDIO RECORDING]
